# IL-33 Is Involved in the Anti-Inflammatory Effects of Butyrate and Propionate on TNFα-Activated Endothelial Cells

**DOI:** 10.3390/ijms22052447

**Published:** 2021-02-28

**Authors:** Meng Li, Betty C. A. M. van Esch, Paul A. J. Henricks, Johan Garssen, Gert Folkerts

**Affiliations:** 1 Division of Pharmacology, Utrecht Institute for Pharmaceutical Sciences, Faculty of Science, Utrecht University, 3584 CG Utrecht, The Netherlands; m.liuu@outlook.com (M.L.); j.garssen@uu.nl (J.G.); g.folkerts@uu.nl (G.F.); 2 Nutricia Research, Immunology, 3584 CT Utrecht, The Netherlands

**Keywords:** short-chain fatty acids, endothelial activation, intracellular IL-33, HDACs, NF-ĸB, MAPK signaling pathways

## Abstract

Short-chain fatty acids (e.g., butyrate and propionate) are able to diminish endothelial cell activation. The aim of this study was to investigate whether intracellular IL-33 mediates the effects of butyrate and propionate on TNFα-induced IL-8 production and vascular cell adhesion molecule-1 (VCAM-1) expression. In addition, it was investigated whether regulating NF-κB and MAPK signaling pathways are involved. Intracellular IL-33 was measured in human endothelial cells (HUVECs) pre-incubated for 24 h with butyrate (0.1 mM or 5 mM), propionate (0.3 mM or 10 mM), or trichostatin A (TSA, 0.5 μM) prior to TNFα (1 ng/mL) stimulation (24 h). The effects of butyrate, propionate, and TSA on TNFα-induced IL-8, vascular cell adhesion molecule-1 (VCAM-1), NF-κB, and MAPK signaling pathways in normal HUVECs and IL-33 siRNA (siIL-33)-transfected HUVECs were compared to study the role of IL-33 in the protective effects of butyrate and propionate. Endogenous IL-33 was highly expressed in the perinuclear in HUVECs, which was significantly reduced by TNFα stimulation. The TNFα-induced reduction in IL-33 was prevented by pre-incubation with butyrate or propionate. Butyrate (0.1 mM), propionate (0.3 mM), and TSA inhibited the IL-8 production and activation of NF-κB. Interestingly, this effect was not observed in siIL-33-transfected HUVECs. The effects of butyrate (5 mM), propionate (10 mM), and TSA (0.5 μM) on VCAM-1 expression and activation of MAPK signaling pathways were not affected by siIL-33 transfection. In conclusion, we showed that the inhibitory effects of butyrate and propionate on TNFα-induced IL-8 production were mediated by the HDACs/IL-33/NF-κB pathway, while their effects on VCAM-1 expression might be associated with the HDACs/MAPK signaling pathway, independently of IL-33.

## 1. Introduction

Atherosclerosis is a chronic inflammatory disease of the vascular system and causes a significant increase in morbidity and mortality of cardiovascular diseases and is initiated by endothelial cell activation [1,2]. The molecular signs of cytokine-induced endothelial activation include the upregulated expression of cellular adhesion molecules, cytokines, and chemokines [3]. Therefore, understanding the precise mechanisms driving endothelial activation may elucidate the pathogenesis of atherosclerosis. The risk of developing atherosclerosis is reduced by consuming a diet high in fiber, possibly due to the production of short-chain fatty acids (SCFAs) [4]. Furthermore, we previously found that the SCFAs butyrate and propionate could improve endothelial cell function by reducing the TNFα-induced production of chemokines (IL-8), vascular cell adhesion molecule-1 (VCAM-1) expression, and peripheral blood mononuclear cell (PBMC) adhesion to endothelial cells via inhibition of histone deacetylases (HDACs) [5,6]. However, the resultant changes downstream following HDAC inhibition are unknown. Studies demonstrated that HDACs, especially HDAC3, regulated IL-33 expression in PBMCs [7,8] and epithelial cells [9]. Trichostatin A (TSA), an HDAC inhibitor, reduced intracellular IL-33 levels, without increasing IL-33 in the serum in lipopolysaccharide-stimulated PBMCs and lung epithelial cells [7,8,9]. However, whether butyrate and propionate improve endothelial cell activation via regulation of HDAC/IL-33 is unknown.

IL-33, a novel member of the IL-1 family of cytokines, is constitutively expressed in the nuclei of endothelial cells from both large and small vessels during homeostasis and released by damaged endothelial cells [10]. IL-33 is thus regarded as an alarm signal that alerts immune cells of tissue damage [10,11]. Immuno-modulatory properties of IL-33 have been studied previously [12], and its role in the modulation of inflammatory pathologies of the respiratory system, gastrointestinal tract, and other inflammatory diseases such as atherosclerosis has also been reported [13,14,15]. Given its prominent involvement in health and disease, a good understanding of IL-33 biology and the mode of action is crucial. IL-33 can be a dual function protein, acting both extracellularly as an IL-1 family cytokine and intracellularly as a nuclear factor regulating gene transcription [16]. As a cytokine, IL-33 binds to ST2 (interleukin-1 receptor-like 1) and induces the activation of endothelial cells towards an inflammatory phenotype by upregulating the expression of inflammatory proteins, including adhesion molecules (VCAM-1), chemokines, and cytokines (IL-6 and IL-8) [13,17,18]. Contrary to its pro-inflammatory effect, extracellular IL-33 also exerts protective effects in the cardiovascular system. For example, IL-33 treatment of ApoE^−/−^ mice alleviates atherosclerosis [19].

Despite these significant findings related to the role of extracellular IL-33, the nuclear function of IL-33 currently remains unclear. Intracellular IL-33 can modulate inflammatory responses by regulating gene expression in two ways: firstly, IL-33 directly localizes to the nuclei and associates with the histones H2A-H2B and chromatin with its chromatin-binding motif being located in its N-terminal region, indicating a critical role for nuclear localization and chromatin association; secondly, nuclear IL-33 interacts with NF-κB [20]. The NF-κB family consists of five members—p65 (or RelA), RelB, c-Rel, p50/p105, and p52/p100—and is involved in the regulation of a variety of physiologic processes [21]. The TNFα-induced activation of NF-κB contributes to the activation of endothelial cells by upregulation of pro-inflammatory cytokines and adhesion molecules in endothelial cells [22]. However, both pro- and anti-inflammatory effects of nuclear IL-33 were reported by either enhancing or reducing NF-κB activity [17,23]. Although IL-33 appears to act as a multifunctional protein in the nucleus, no study has been performed to demonstrate whether endogenous IL-33 is involved in the effects of butyrate and propionate on the regulation of TNFα-induced endothelial activation via modulating activation of NF-ĸB. Moreover, TNFα also induces activation of the MAPK signaling pathways, including ERK1/2, JNK, and p38MAPK, which are also involved in regulation the expression of adhesion molecules [24,25,26]. Normally, activation of JNK and p38MAPK signaling pathways are essential for VCAM-1 expression in endothelial cells [27,28,29,30]. SCFAs can modulate the MAPK signaling pathways by inhibiting HDAC activity in immune cells [31], but the modulating mechanisms of intracellular pathways in endothelial cells are currently unknown. In this study, we investigated which intracellular pathways were involved in the HDAC-regulated anti-inflammatory responses of butyrate and propionate in TNFα-induced endothelial activation. It was found that the HDACs/IL-33/NF-ĸB signaling cascade was involved in the effects of butyrate and propionate on IL-8 production, and HDACs/MAPK signaling pathways were involved in their effects on VCAM-1 expression. These findings offer a better understanding of the anti-inflammatory effects of butyrate and propionate on TNFα-induced endothelial activation.

## 2. Results

### 2.1. IL-33 Was Expressed in the Cytoplasm and Perinuclear in Human Endothelial Cells (HUVECs)

**Figure 1:**
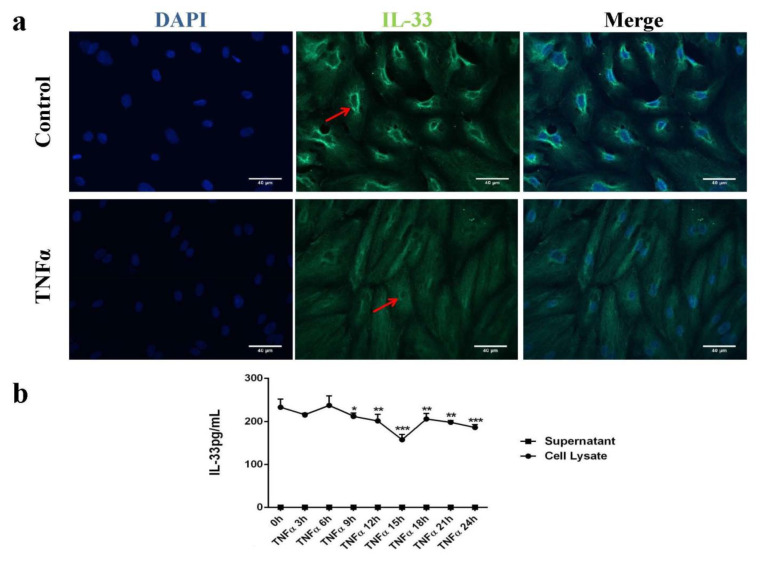
The effects of TNFα on IL-33 expression in human endothelial cells (HUVECs). (**a**) Representative images of DAPI and IL-33 duplicate fluorescence staining showing intracellular IL-33 expression and location in untreated (control) and TNFα (1 ng/mL)-treated HUVECs. Cell nucleus was visualized by a blue signal, and IL-33 was visualized by a green signal. Red arrows indicate high expression of IL-33 near the nuclear membrane in normal cells, which was decreased after 24 h of TNFα stimulation of HUVECs. Scale bar is 40 µm. (**b**) ELISA analysis of the effect of TNFα stimulation on expression of intra- and extracellular IL-33 during 0-24 h of incubation. *: *p* < 0.05, **: *p* < 0.01, ***: *p* < 0.001 compared with the control at 0 h (*n* = 4).

Under non-stimulated conditions, IL-33 was expressed in the cytoplasm and perinuclear. IL-33 expression was high near the nuclear membrane (Figure 1a) and was decreased after TNFα stimulation (Figure 1a). Human endothelial cells (HUVECs) were treated with TNFα (1 ng/mL), and supernatants and cell lysates were harvested every 3 h for a period of 24 h. IL-33 concentrations in cell lysates were significantly reduced after 6 h of TNFα stimulation and lasted for 24 h. In contrast, although the proteins in the supernatant were concentrated 10 times, IL-33 was not detectable in the supernatant (Figure 1b), which indicated that TNFα treatment did not result in the extracellular release of IL-33, nor was apoptosis induced.

### 2.2. Butyrate and Propionate Prevented a TNFα-Induced Decrease in the Intracellular IL-33 Level

**Figure 2:**
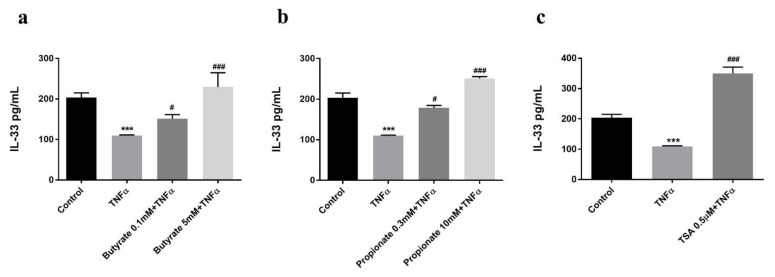
The effects of (**a**) butyrate, (**b**) propionate, and (**c**) TSA on the expression of endogenous IL-33 in HUVECs. TNFα treatment significantly decreased intracellular IL-33 level, which was prevented by butyrate, propionate, and TSA treatments. *** *p* < 0.001 compared with the control group; ^#^: *p* < 0.05, ^###^: *p* < 0.001 compared with the TNFα group (*n* = 4).

The TNFα-induced decrease in intracellular IL-33 concentration after 24 h of stimulation was prevented when the HUVECs were pre-incubated for 24 h with butyrate (0.1 or 5 mM) or propionate (0.3 or 10 mM) (Figure 2a,b). TSA (0.5 µM), the HDAC inhibitor, showed a similar effect (Figure 2c).

### 2.3. siIL-33 Transfection

**Figure 3:**
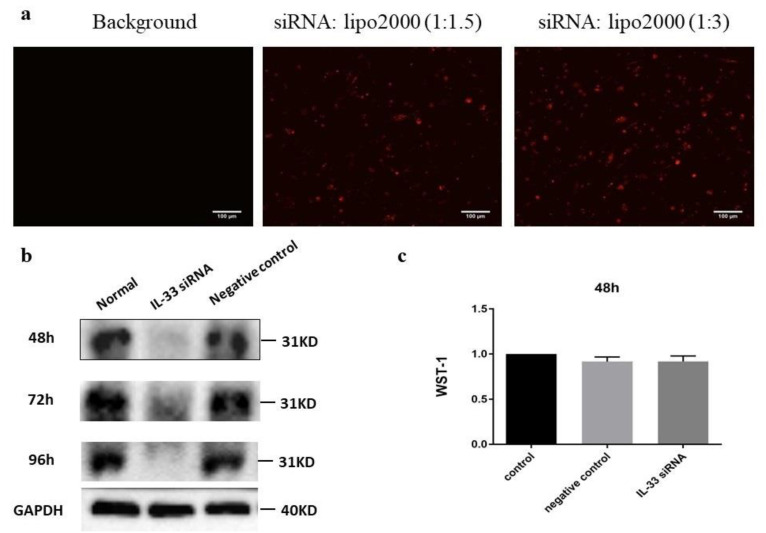
siIL-33 transfection in HUVECs. (**a**) Transfection efficiency test. HUVECs were transfected with different ratios of Block-iT and lipo-2000 for 6 h. Incubation with only fluorescence labeled Block-iT without transfection reagent was regarded as the background. Scale bar is 100 µm. (**b**) Western blot analysis of confluent primary HUVEC lysates was performed using antibodies against IL-33 and GAPDH. IL-33 expression was silenced by siRNA transfection after 48, 72, and 96 h. The controls are untreated normal HUVECs. In a silencer negative control, IL-33 expression was not affected. (**c**) After HUVECs were transfected by IL-33 siRNA for 48 h, the WST-1 test was performed in control (untreated), silencer negative, and IL-33 siRNA-transfected cells to indicate cell viability and showed no influence on cell viability (*n* = 6).

IL-33 expression was silenced in HUVECs by siRNA transfection. The weight ratio of 1:3 between siRNA and lipo-2000 produced a higher transfection efficiency than a weight ratio of 1:1.5 (Figure 3a). The expression level of IL-33 protein was then measured by western blot after transfection with IL-33 siRNA for 48, 72, and 96 h. IL-33 expression was efficiently suppressed by siIL-33 transfection compared with the negative control at all three time points (Figure 3b). All experiments that were performed with siIL-33-transfected HUVECs started from 48 h after transfection. We therefore performed a WST-1 test to measure cell proliferation and viability at 48 h after siIL-33 transfection, and found no effect of siIL-33 transfection on cell proliferation and viability (Figure 3c).

### 2.4. The Inhibition of TNFα-Induced IL-8 Production by Butyrate and Propionate Is IL-33-Dependent

**Figure 4:**
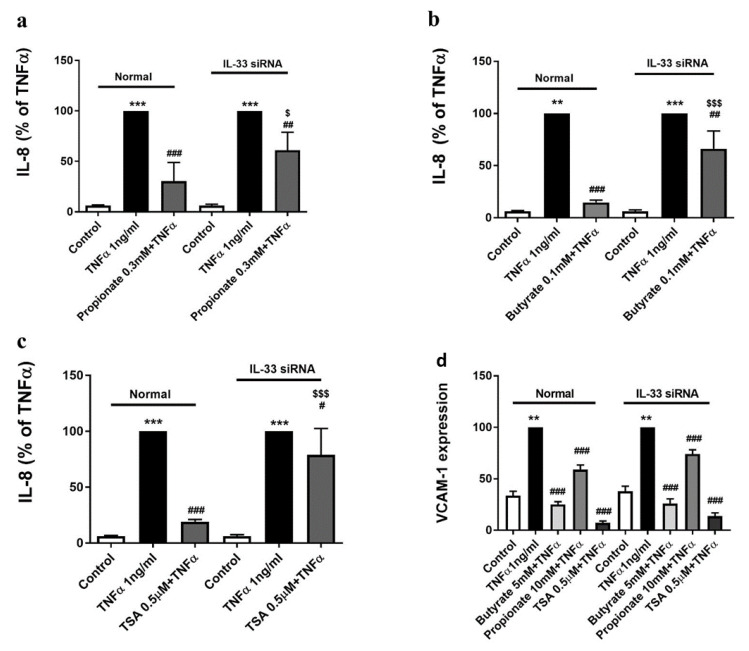
The effects of propionate and butyrate on TNFα-stimulated IL-8 production (24 h) and VCAM-1 expression (8 h) in normal and siIL-33-transfected HUVECs. (**a**) Butyrate (0.1 mM) treatment significantly reduced TNFα-induced IL-8 production in normal HUVECs, which was significantly restored in siIL-33-transfected HUVECs. (**b**,**c**) Similar effects were found in cells treated with propionate (0.3 mM) and TSA (0.5 µM). (**d**) In siIL-33-transfected HUVECs, TNFα-increased VCAM-1 expression was inhibited by butyrate (5 mM), propionate (10 mM), and TSA (0.5 µM) treatments, which were similar in normal HUVECs. **: *p* < 0.01, ***: *p* < 0.001 compared with the control group; ^#^: *p* < 0.05, ^##^: *p* < 0.01, ^###^: *p* < 0.001 compared with the TNFα group; ^$^: *p* < 0.05, ^$$$^: *p* < 0.001 compared with the effects of butyrate, propionate, or TSA treatment in normal HUVECs (*n* = 4).

The IL-8 production induced by TNFα-induced HUVECs was suppressed by butyrate (0.1 mM) and propionate (0.3 mM; Figure 4a,b). These inhibitory effects of butyrate and propionate on IL-8 production were significantly prevented in siIL-33-transfected HUVECs (Figure 4a,b). Similar results were obtained by pre-incubation with the HDAC inhibitor, TSA (0.5 µM; Figure 4c).

### 2.5. The Inhibition of the TNFα-Induced VCAM-1 Expression by Butyrate and Propionate Is IL-33-Independent

The VCAM-1 expression induced on TNFα-stimulated HUVECs was suppressed by butyrate and propionate (Figure 4d). In contrast to the IL-8 production, the inhibitory effects of butyrate and propionate on VCAM-1 expression were not different in siIL-33-transfected HUVECs (Figure 4d). TSA pre-incubation also blocked VCAM-1 expression in siIL-33-transfected HUVECs (Figure 4d).

### 2.6. The Butyrate and Propionate-Inhibited NF-κB Activation Is IL-33 Dependent

**Figure 5:**
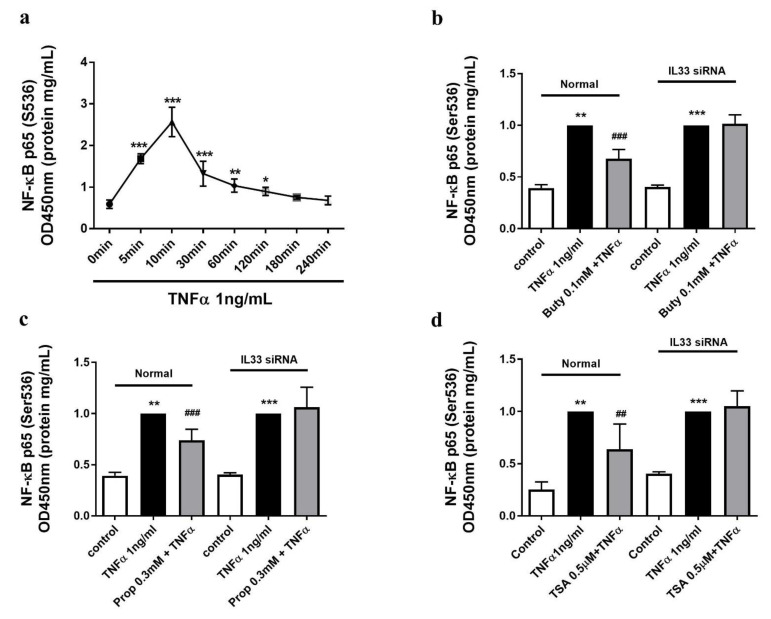
Butyrate- and propionate-inhibited activation of NF-κB was IL-33-dependent. (**a**) TNFα induced phosphorylation of p65 in a time-dependent manner for 240 min (*n* = 4). (**b**) Butyrate (0.1 mM) treatment inhibited a TNFα-induced (p)p65 level, which was absent in IL-33 siRNA-transfected HUVECs. (**c**) Similar effects were obtained in propionate-treated groups. (**d**) TSA (0.5 μM) also inhibited p65 activation, and its effect was absent in siIL-33-transfected HUVECs. *: *p* < 0.05, **: *p* < 0.01, ***: *p* < 0.001 compared with the control group; ^##^: *p* < 0.01, ^###^: *p* < 0.001 compared with the TNFα group (*n* = 4).

The phosphorylation level of p65, the subunit of NF-κB, was induced by TNFα in a time-dependent manner and reached a maximum after 10 min of stimulation (Figure 5a). Pre-incubation of normal HUVECs with a low concentration of butyrate (0.1 mM) and propionate (0.3 mM) inhibited TNFα-induced (10 min) phosphorylation of p65, which was absent in siIL-33-transfected HUVECs (Figure 5b,c). High concentrations of butyrate (5 mM) and propionate (10 mM) showed no effect on TNFα-induced (p)p65 levels (data not shown). Furthermore, TSA treatment also showed inhibitory effects on the phosphorylation of p65 in normal cells, which was absent in siIL-33-transfected HUVECs (Figure 5d).

### 2.7. Butyrate and Propionate Modulated the MAPK Signaling Pathway Independently of IL-33

**Figure 6:**
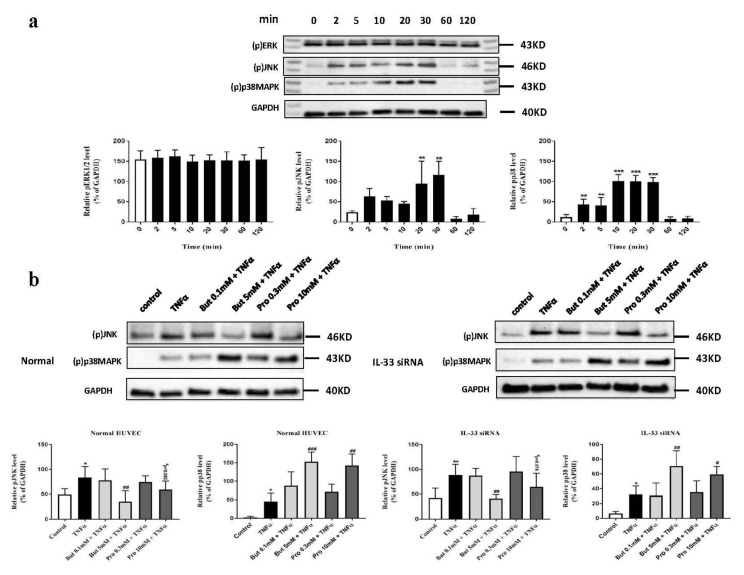

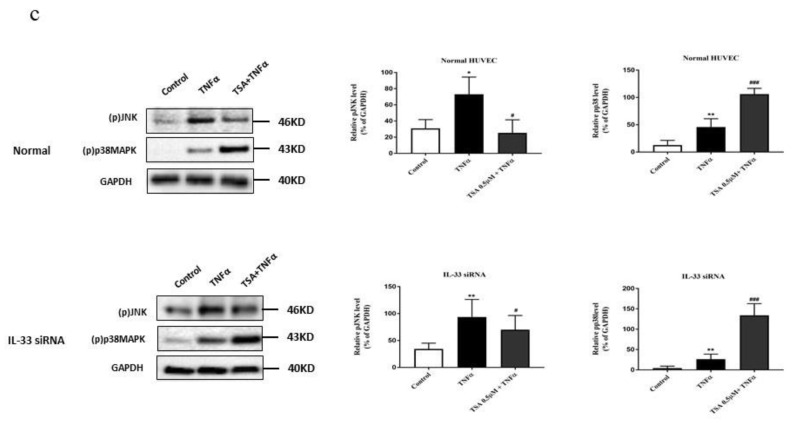
The effects of butyrate and propionate on TNFα-induced MAPK signaling pathways activation in normal and siIL-33-transfected HUVECs. (**a**) Western blot analysis demonstrated time-dependent activation of JNK and p38MAPK, but not ERK1/2, by TNFα in HUVECs. (**b**) Butyrate (5 mM) and propionate (10 mM) inhibited activation of JNK, but facilitated activation of p38MAPK in normal HUVECs, which were not affected by IL-33 transfection. (**c**) TSA (0.5 μM) showed similar effects with butyrate and propionate on activation of JNK and p38MAPK. *: *p* < 0.05, **: *p* < 0.01, ***: *p* < 0.001 compared with the control group; ^#^: *p* < 0.05, ^##^: *p* < 0.01, ^###^: *p* < 0.001 compared with the TNFα group (*n* = 3).

To investigate whether the MAPK signaling pathways are also affected by butyrate and propionate treatment and whether IL-33 modulates activation of the MAPK signaling pathways, phosphorylation levels of MAPK family proteins, including ERK1/2, JNK, and p38MAPK, in normal and siIL-33-transfected HUVECs were investigated by western blot. Phosphorylation of JNK and p38MAPK, but not ERK1/2, was activated by TNFα stimulation in a time-dependent manner (Figure 6a), with phosphorylation levels of JNK and p38MAPK peaking around 20–30 min (Figure 6a). The effects of butyrate (0.1 and 5 mM) and propionate (0.3 and 10 mM) on protein levels of (p)JNK and (p)p38MAPK induced by 30 min of TNFα stimulation were then investigated. In normal HUVECs, low concentrations of butyrate (0.1 mM) and propionate (0.3 mM) had no significant effects on phosphorylation of JNK and p38MAPK (Figure 6b). However, high concentrations of butyrate (5 mM) and propionate (10 mM) inhibited increased levels of (p)JNK, but increased the protein level of (p)p38MAPK (Figure 6b). These effects were not changed in siIL-33-transfected HUVECs (Figure 6b). TSA showed similar effects as high concentrations of butyrate and propionate on the phosphorylation of JNK and p38MAPK, both in normal and siIL-33-transfected HUVECs (Figure 6c).

## 3. Discussion

Accumulating evidence indicates that endothelial activation is an early marker for atherosclerosis [32], which is characterized by increased production of cytokines and chemokines and the expression of adhesion molecules [3]. In a previous study, we showed that TNFα-induced endothelial activation was diminished by SCFA, especially butyrate and propionate, via inhibition of HDAC activity [6]. HDACs regulate the expression of nuclear IL-33, which is involved in inflammatory response in immune cells by regulating NF-κB and MAPK signaling pathways [9,23]. However, the molecular and downstream mechanisms underlying HDAC inhibition by butyrate and propionate are completely unknown in endothelial cells. Therefore, we investigated whether nuclear IL-33 mediates the beneficial effects of butyrate and propionate on endothelial activation by regulation of NF-κB or MAPK signaling pathways. In the present study, we demonstrated several novel findings. First, we found that IL-33 was located in the cytoplasm, mainly near the nucleus. This IL-33 was significantly decreased by TNFα stimulation and restored by butyrate and propionate. Moreover, IL-33 was not detectable in the supernatant with or without TNFα treatment. Second, the inhibitory effects of butyrate and propionate on IL-8 production were IL-33-dependent, while their effects on VCAM-1 expression were not. Third, low concentrations of butyrate (0.1 mM) and propionate (0.3 mM), which affected IL-8 production, inhibited phosphorylation of p65(S536) in an IL-33-dependent way. Fourth, high concentrations of butyrate (5 mM) and propionate (10 mM), which affected TNFα-induced VCAM-1 expression, inhibited activation of the JNK pathway and facilitated activation of the p38MAPK pathway in an IL-33-independent way. A limitation of our study is that we used normal HUVECs as a control for the IL-33 silenced HUVECs. Future studies are needed to exclude any non-specific effects of siRNA delivery. Finally, TSA (an HDAC inhibitor) showed effects similar to those of butyrate and propionate on IL-8 production, VCAM-1 expression, and activation of NF-κB and MAPK signaling pathways in normal and siIL-33-transfected HUVECs. Understanding the underlying mechanisms of action by butyrate and propionate on endothelial activation offers a novel therapeutic strategy in the treatment of atherosclerosis.

IL-33, belonging to the IL-1 family, can function both as a ligand for ST2 and as a nuclear factor to modulate inflammatory responses [11,17,33]. IL-33 is abundantly expressed in the nuclei of endothelial cells during homeostasis and can be induced or suppressed by different kinds of stimulation [7,10,34]. So far, IL-33 expression at the mRNA and protein level in endothelial cells has been shown in vivo [10]. In contrast, the expression of IL-33 in primary HUVECs has not been reported. In the present study, we found IL-33 constitutively expressed in the cytoplasm and nucleus of HUVECs. Moreover, IL-33 was concentrated near the nucleus, which was reduced by TNFα treatment. As IL-33 was not detected in the supernatant of normal or TNFα-stimulated HUVECs, we can infer it was behaving as a nuclear factor in this study. The TNFα-inhibited IL-33 expression was prevented by butyrate and propionate treatment, which might be associated with their inhibitory effects on HDAC activity. HDACs, especially HDAC3, are involved in the regulation of endogenous IL-33 levels in PBMCs, and the inhibition of HDACs by TSA reduced IL-33 in LPS-stimulated PBMCs [8]. In our previous study, HDAC3 was highly expressed in HUVECs, and butyrate or propionate inhibited HDAC activity [6]. In order to further support the hypothesis that butyrate- and propionate-modulated endogenous IL-33 expression was mediated by the inhibition of HDAC activity, the effects of butyrate/propionate and TSA, a potent HDAC inhibitor, on IL-33 expression were compared. Interestingly, TSA increased intracellular IL-33 expression and had effects similar to those of butyrate and propionate. These compounds even increased IL-33 levels in the presence of TNFα above control levels. A possible mechanism could be that the basal IL-33 production (without any stimulation) is already under the control of HDACs and that the SCFA and TSA increase the basal production of IL-33 via inhibition of the basal HDAC activity. All these data suggested IL-33 was regulated by HDACs and acted as a nuclear factor rather than as a cytokine under this experiment set-up and might be involved in the effects of butyrate and propionate on the regulation of TNFα-induced endothelial activation.

IL-8 is a prominent chemokine released by endothelial cells, which triggers the recruitment of immune cells to the atherosclerotic sites and mediates inflammatory responses [35]. VCAM-1 expression can be induced by TNFα and mediates monocyte adhesion and transmigration [36,37]. NF-κB regulates the TNFα-induced inflammatory response in endothelial cells, including the production of pro-inflammatory cytokines such as IL-8 and the expression of adhesion molecules such as VCAM-1 [38,39]. Therefore, inhibition of NF-κB can dampen TNFα-induced endothelial activation. IL-33 functions as a nuclear factor with pro- and anti-inflammatory properties by regulating the activation of NF-κB [20]. Nuclear IL-33 can function as a direct transcriptional activator of NF-κB that upregulates the basal expression of NF-κB p65 showing pro-inflammatory properties [17]. Nuclear IL-33 can also interact with the p65 and reduce p65 binding to its cognate DNA and dampen NF-κB-stimulated gene transcription, leading to anti-inflammatory responses [23]. In the present study, we found the reduced phosphorylation of p65 and IL-8 production by butyrate (0.1 mM) and propionate (0.3 mM) was IL-33-dependent. p65 is the specific subunit of NF-ĸB that can bind to the IL-8 promoter to regulate IL-8 expression [40]. Therefore, we can predict that nuclear IL-33 was involved in the inhibitory effects of butyrate and propionate on IL-8 production by interacting with p65 and reducing p65 binding to the IL-8 promoter, explaining the molecular mechanism behind the anti-inflammatory properties of the SCFA. In contrast, butyrate (5 mM) and propionate (10 mM) showed no effects on the activation of NF-ĸB p65, and their inhibitory effects on VCAM-1 expression were not changed in siIL-33-transfected HUVECs. This indicates that the effect of butyrate and propionate on VCAM-1 expression was IL-33- and NF-κB-independent, thus mediated by another mechanism. In previous studies, the activation of JNK [27,41] and p38MAPK signaling pathways [28,30] was involved in the modulation of VCAM-1 expression. In this study, JNK and p38MAPK pathways, but not ERK1/2, were activated by TNFα, and subsequently regulated by butyrate and propionate. This indicates the involvement of JNK and p38MAPK signaling pathways in the effects of butyrate and propionate on VCAM-1 expression. Interestingly, we found that butyrate (5 mM) and propionate (10 mM) inhibited the activation of JNK but facilitated the activation of the p38MAPK pathway, independently of IL-33. Despite the opposite effects on the activation of JNK and p38MAPK, butyrate and propionate inhibited VCAM-1 expression in TNFα-induced endothelial cells. We can only speculate that, although both JNK and p38MAPK signaling pathways are upregulated by TNFα stimulation and involved in regulating VCAM-1 expression, pJNK might be more important in mediating the effects of butyrate on VCAM-1 expression [41,42]. Moreover, TSA showed effects similar to those of butyrate and propionate on VCAM-1 expression and activation/inhibition of JNK and p38MAPK. Therefore, the inhibitory effects of butyrate or propionate on VCAM-1 expression might be mediated by HDACs/JNK or the p38MAPK signaling cascade.

In conclusion, this is the first study demonstrating the involvement of nuclear IL-33 in the anti-inflammatory effects of butyrate and propionate on TNFα-activated endothelial cells, and IL-33 as a nuclear factor showed anti-inflammatory properties. Furthermore, the effects of butyrate and propionate on IL-8 production were mediated by the HDAC/IL-33/NF-κB pathway, while their effects on VCAM-1 expression were associated with the IL-33-independent HDAC/MAPK signaling pathways. These findings provide a better understanding of the beneficial effects of butyrate and propionate on TNFα-induced endothelial activation, and may lead to novel protective pathways in the prevention of endothelial activation and opportunities for therapeutic intervention.

## 4. Materials and Methods

### 4.1. Materials

Sodium butyrate, propionate, TSA, WST-1, and a protease inhibitor cocktail were purchased from Sigma-Aldrich. Butyrate and propionate were dissolved in endothelial growth factor (EGM-2) and TSA in DMSO and then further diluted in EGM-2. Concentrations used were based on recent publications of our group [5,6]. Human recombinant TNFα was bought from eBioscience. The human IL-8 enzyme-linked immunosorbent assay (ELISA) kit, Lipofectamine 2000 (lipo-2000), and the BLOCK-iT Alexa Fluor Red Fluorescent control were purchased from Invitrogen. The NF-κB p65 (pS536) ELISA kit was bought from Cell signaling technology. The IL-33 ELISA kit was purchased from U-CyTech biosciences. The IL-33 monoclonal antibody and Sliencer GAPDH siRNA (human) were bought from Thermo Fisher Scientific. Silencer pre-designed on-Targetplus SMARTpool siRNA IL-33 and siGENOME Non-Targeting siRNA pool (Silencer negative control) were bought from Dharmacon. The following monoclonal antibodies: the anti-phospho p38 (phospho T180 + Y182) antibody, the anti-JNK1+JNK2 (phospho T183 + Y185) antibody, the anti-ERK1/2 (phospho Thr202/Tyr204) antibody, the anti-GAPDH antibody, the rabbit anti-mouse IgG H&L (HRP) conjugated antibody, and the goat anti-rabbit IgG H&L (HRP) conjugated antibody were purchased from Cell Signaling Technology and Abcam.

### 4.2. HUVEC Culture

HUVECs were isolated from an umbilical vein by adapting the method of Jaffe et al. [43] and were kindly provided by J.H. van Kats-Renaud (University Medical Center, Utrecht). Informed consent was obtained from all subjects and was provided in accordance with the Declaration of Helsinki. Approval was obtained from the medical ethics committee of the University Medical Center Utrecht (Utrecht, The Netherlands). HUVECs were cultured in EGM-2 (Lonza) containing 2% fetal bovine serum and VEGF for rapid proliferation. HUVEC cultures were maintained in a humidified incubator at 37 °C and 5% CO_2_, and medium was changed every 2–3 days. Passages 2–7 of HUVECs were used.

### 4.3. Small Interfering RNA (siRNA) and Cytotoxicity Test

Silencer pre-designed on-Targetplus SMARTpool siRNA IL-33 and the siGENOME Non-Targeting siRNA pool (Silencer negative control) were used. siRNA transfection was performed as described previously [13]. HUVECs were seeded on 96-well plates and 6 well-plates at 1.25 × 10^4^ and 2 × 10^5^ cells/well, respectively, until cells reached 60–80% confluence. First, a siRNA transfection efficiency test in HUVECs was performed. HUVECs were incubated with a mixture of fluorescence labeled siRNA (Block-iT) in 96-well plates, at a final concentration of 50 nM, and different amounts of lipo-2000 according to the manufacturer’s instructions. After 6 h of transfection, HUVECs were washed with PBS and cultured in a regular growth medium. Fluorescence images were collected. Secondly, HUVECs in 6-well plates were transfected with IL-33 siRNA along with lipo-2000, or with silencer negative control siRNA, which has no significant sequence similarity to human gene sequences combined with lipo-2000. siRNA (10 μL of stock solution at 10 μM) and oligofectamine (10 μL) were mixed in 0.4 mL Opti-MEM-1 for 20 min at room temperature before addition to the cells in Opti-MEM-1 medium (2 mL final volume). After 6 h of transfection, the medium was replaced with a regular growth medium. The protein expression level of intracellular IL-33 was measured at 48, 72, and 96 h after the start of transfection by western blot. Lastly, a WST-1 test in 96-well plates was performed to measure cell proliferation and viability after 48 h of siIL-33 transfection and was done according to the manufacturer’s protocol.

### 4.4. Immunocytochemistry

HUVECs were seeded in 96-well plates and incubated at 37 °C and 5% CO_2_ for two days. Cells were treated with or without TNFα (1 ng/mL) for 24 h and then processed for immunocytochemical analysis as previously described [44]. Briefly, cells were first treated with a permeabilization solution (0.25% triton-X) for 10 min and then washed with cold PBS. Cells were then incubated with a blocking buffer for 1 h and then washed with cold PBS. Next, cells were stained with IL-33 primary antibodies (1:300) for 1 h at room temperature and with a goat anti-rabbit Alexa Fluor 568 second antibody (1:400) for 1 h. The nuclei of cells were stained by exposure to DAPI at a concentration of 300 nM for 5 min. The negative controls were stained only with the second antibody. The images of stained cells were collected by a Yokogawa CV7000S imager.

### 4.5. IL-33 ELISA Assay

Cells were treated with TNFα for 24 h, and supernatants and cell lysates were collected every 3 h to determine the appropriate stimulation time. Proteins in supernatants were concentrated and re-suspended in an ELISA assay buffer with a protease inhibitor. Cells lysates were collected by splitting cells with a cell lysis buffer containing a protease inhibitor. IL-33 concentration was measured in supernatants and cell lysates by ELISA according to the manufacturer’s instructions by using a standard curve. The optical densities of samples were detected using a microplate reader at a wavelength of 450 nm. Cells were then pre-treated with butyrate (0.1 and 5 mM), propionate (0.3 and 10 mM) or TSA (0.5 μM) for 24 h and then treated with TNFα (1 ng/mL) for 24 h. The endogenous IL-33 concentration was assayed by ELISA.

### 4.6. IL-8 Production in siIL-33 Transfected HUVEC

siIL-33-transfected HUVECs were pre-incubated with butyrate (0.1 mM), propionate (0.3 mM), or TSA (0.5 μM) for 24 h, followed by TNFα (1 ng/mL) stimulation for 24 h. The supernatants were collected and stored at −20 °C for later analysis. The IL-8 concentration in the supernatant was measured by ELISA according to the manufacturer’s instructions.

### 4.7. VCAM-1 Expression in siIL-33 Transfected HUVECs by Flow Cytometry

After transfection with IL-33 siRNA for 48 h, HUVECs were pre-incubated with butyrate (5 mM), propionate (10 mM), or TSA (0.5 µM) for 24 h followed by 8 h of TNFα (1 ng/mL) stimulation. Cells were then stained with a human VCAM-1 PE-conjugated antibody and cell viability dye according to the manufacturer’s protocol and then detached from the culture plates with 0.05% trypsin-EDTA. VCAM-1 expression on siIL-33-transfected HUVECs was measured by a flow cytometer (FACSCanto II), and data were analyzed by Flowlogic, version 7.

### 4.8. Activation of NF-κB P65 in Normal and siIL-33 Transfected HUVEC

HUVECs were first treated with TNFα (1 ng/mL) for 4 h, and cell lysates were collected at 0, 5, 10, 30, 60, 120, 180, and 240 min to determine the optimal stimulation time. Normal and siIL-33-transfected HUVECs were then pre-incubated with butyrate (0.1 and 5 mM), propionate (0.3 and 10 mM), or TSA (0.5 µM) for 24 h, followed by 10 min of TNFα stimulation. Cell lysates were collected in a cell lysis buffer containing protease inhibitors, and samples were stored at −80 °C. The phosphorylation level of p65 (S536) in the cell lysate was measured by ELISA according to the manufacturer’s instructions.

### 4.9. Activation of MAPK Signaling Pathway

Again, HUVECs were treated with TNFα (1 ng/mL) for 2 h, and cell lysates were collected at 0, 2, 5, 10, 20, 30, 60, and 120 min to determine the optimal stimulation time. Normal and siIL-33-transfected HUVECs were then pre-incubated with butyrate (0.1 and 5 mM), propionate (0.3 and 10 mM), or TSA (0.5 µM) for 24 h, followed by 30 min of TNFα stimulation. Cells were lysed in RIPA buffer containing protease and phosphatase inhibitor. Proteins were separated on 15% polyacrylamide gel and transferred to a methanol-activated PVDF membrane. The membrane was blocked for 1 h in 5% milk and then incubated with primary antibodies at 4 °C overnight. Secondary antibody incubation was performed at room temperature for 1 h. Primary antibodies used were as follows: the anti-phospho p38 (phospho T180 + Y182; 1:1000) antibody, the anti-JNK1+JNK2 (phospho T183 + Y185; 1:1000) antibody, the anti-ERK1/2 (phospho Thr202/Tyr204; 1:1000) antibody, and the anti-GAPDH antibody (1:1000). Secondary antibodies used were the goat anti-rabbit IgG H&L (HRP) conjugated antibody (1:10,000) and the rabbit anti-mouse IgG H&L (HRP) conjugated antibody (1:10,000). Protein expression was quantified by an assessment of the optical density of protein bands and standardized against GAPDH protein expression with the use of ImageLab software.

### 4.10. Statistical Analysis

The data and statistical analysis comply with the recommendations for experimental design and analysis in pharmacology [45]. All data are expressed as mean ± S.D. Group comparisons were performed with one-way ANOVA using post hoc Bonferroni correction. SPSS was used for all statistical analyses. In all cases, *p* values < 0.05 were considered significant.

## Data Availability

Raw data are stored at Utrecht University. The dataset used and/or analyzed during the current study are available from the corresponding author on reasonable request.

## References

[1] Singh R.B., Mengi A.S., Xu Y.-J., Arneja A.S., Dhalla N.S. (2002). Pathogenesis of atherosclerosis: A multifactorial process. Exp. Clin. Cardiol..

[2] Deanfield J.E., Halcox J.P., Rabelink T.J. (2007). Endothelial function and dysfunction: Testing and clinical relevance. Circulation.

[3] Hansson G.K. (2005). Inflammation, Atherosclerosis, and Coronary Artery Disease. N. Engl. J. Med..

[4] Ohira H., Tsutsui W., Fujioka Y. (2017). Are Short Chain Fatty Acids in Gut Microbiota Defensive Players for Inflammation and Atherosclerosis?. J. Atheroscler. Thromb..

[5] Li M., Van Esch B.C., Henricks P.A.J., Garssen J., Folkerts G. (2018). Time and Concentration Dependent Effects of Short Chain Fatty Acids on Lipopolysaccharide- or Tumor Necrosis Factor α-Induced Endothelial Activation. Front. Pharmacol..

[6] Li M., van Esch B.C., Henricks P.A.J., Garssen J., Folkerts G. (2018). The anti-inflammatory effects of short chain fatty acids on lipopolysaccharide- or tumor necrosis factor α-stimulated endothelial cells via activation of GPR41/43 and inhibi-tion of HDACs. Front. Pharmacol..

[7] Kuchler A.M., Pollheimer J., Balogh J., Sponheim J., Manley L., Sorensen D.R., De Angelis P.M., Scott H., Haraldsen G. (2008). Nuclear interleukin-33 is generally expressed in resting endothelium but rapidly lost upon angiogenic or proinflamma-tory activation. Am. J. Pathol..

[8] Zhang F., Tossberg J.T., Spurlock C.F., Yao S., Aune T.M., Sriram S. (2014). Expression of IL-33 and its epigenetic regulation in multiple sclerosis. Ann. Clin. Transl. Neurol..

[9] Toki S., Goleniewska K., Reiss S., Zhou W., Newcomb D.C., Bloodworth M.H., Stier M.T., Boyd K.L., Polosukhin V.V., Subramaniam S. (2016). The histone deacetylase inhibitor trichostatin A suppresses murine innate allergic in-flammation by blocking group 2 innate lymphoid cell (ILC2) activation. Thorax.

[10] Moussion C., Ortega N., Girard J.-P. (2008). The IL-1-Like Cytokine IL-33 Is Constitutively Expressed in the Nucleus of Endothelial Cells and Epithelial Cells In Vivo: A Novel ‘Alarmin’?. PLoS ONE.

[11] Cayrol C., Girard J.-P. (2018). Interleukin-33 (IL-33): A nuclear cytokine from the IL-1 family. Immunol. Rev..

[12] Duan L., Chen J., Zhang H., Yang H., Zhu P., Xiong A., Xia Q., Zheng F., Tan Z., Gong F. (2012). Interleukin-33 Ameliorates Experimental Colitis through Promoting Th2/Foxp3+ Regulatory T-Cell Responses in Mice. Mol. Med..

[13] Gautier V., Cayrol C., Farache D., Roga S., Monsarrat B., Burlet-Schiltz O., de Peredo A.G., Girard J.-P. (2016). Extra-cellular IL-33 cytokine, but not endogenous nuclear IL-33, regulates protein expression in endothelial cells. Sci. Rep..

[14] Montanari E., Stojkovic S., Kaun C., Lemberger C.E., de Martin R., Rauscher S., Gröger M., Maurer G., Neumayer C., Huk I. (2016). Interleukin-33 stimulates GM-CSF and M-CSF production by human endothe-lial cells. Thromb. Haemost..

[15] Schwartz C., O’Grady K., Lavelle E.C., Fallon P.G. (2016). Interleukin 33: An innate alarm for adaptive responses beyond Th2 immunity-emerging roles in obesity, intestinal inflammation, and cancer. Eur. J. Immunol..

[16] Miller A.M. (2011). Role of IL-33 in inflammation and disease. J. Inflamm..

[17] Choi Y.S., Park J.A., Kim J., Rho S.S., Park H., Kim Y.M., Kwon Y.G. (2012). Nuclear IL-33 is a transcriptional regulator of NF-κB p65 and induces endothelial cell activation. Biochem. Biophys. Res. Commun..

[18] Demyanets S., Konya V., Kastl S.P., Kaun C., Rauscher S., Niessner A., Pentz R., Pfaffenberger S., Rychli K., Lem-berger C.E. (2011). Interleukin-33 induces ex-pression of adhesion molecules and inflammatory activation in human endothelial cells and in human atherosclerotic plaques. Arterioscler. Thromb. Vasc. Biol..

[19] Miller A.M., Xu D., Asquith D.L., Denby L., Li Y., Sattar N., Baker A.H., McInnes I.B., Liew F.Y. (2008). IL-33 reduces the development of atherosclerosis. J. Exp. Med..

[20] Ohta S., Tago K., Funakoshi-Tago M., Matsugi J., Yanagisawa K. (2016). Intracellular NF-HEV/IL-33 harbors essential roles in Ras-induced cellular transformation by contributing to cyclin D1 protein synthesis. Cell. Signal..

[21] Oeckinghaus A., Ghosh S. (2009). The NF-κB family of transcription factors and its regulation. Cold Spring Harb. Perspect. Biol..

[22] Zheng X.-X., Zhou T., Wang X.-A., Tong X.-H., Ding J.-W. (2015). Histone deacetylases and atherosclerosis. Atherosclerosis.

[23] Ali S., Mohs A., Thomas M., Klare J., Ross R., Schmitz M.L., Martin M.U. (2011). The dual function cytokine IL-33 interacts with the transcription factor NF-κB to dampen NF-κB-stimulated gene transcription. J. Immunol..

[24] Pietersma A., Tilly B.C., Gaestel M., De Jong N., Lee J.C., Koster J.F., Sluiter W. (1997). P38 Mitogen Activated Protein Kinase Regulates Endothelial VCAM-1 Expression at the Post-transcriptional Level. Biochem. Biophys. Res. Commun..

[25] Hubbard A.K., Rothlein R. (2000). Intercellular adhesion molecule-1 (ICAM-1) expression and cell signaling cascades. Free. Radic. Biol. Med..

[26] Ueno H., Pradhan S., Schlessel D., Hirasawa H., Sumpio B.E. (2006). Nicotine enhances human vascular endothelial cell expres-sion of ICAM-1 and VCAM-1 via protein kinase C, p38 mitogen-activated protein kinase, NF-κB, and AP-1. Cardiovasc. Toxicol..

[27] Li M.S., Rafiee P., Fisher P.J., Lamirand T.H., Taras A.R., Shidham V., Johnson C.P., Binion D.G. (2000). Vascular cell adhe-sion molecule-1 (VCAM-1) expression in human intestinal micro-vascular endothelial cells (HIMEC) is mediated by JNK, intracellular oxyradicals and NFκB activation. Gastroenterology.

[28] Ono H., Ichiki T., Ohtsubo H., Fukuyama K., Imayama I., Iino N., Masuda S., Hashiguchi Y., Takeshita A., Sunagawa K. (2006). cAMP-response element-binding protein mediates tumor necrosis factor-α-induced vascular cell adhesion molecule-1 expression in endothelial cells. Hypertens. Res..

[29] Roussel L., Houle F., Chan C., Yao Y., Bérubé J., Olivenstein R., Martin J.G., Huot J., Hamid Q., Ferri L. (2010). IL-17 Promotes p38 MAPK-Dependent Endothelial Activation Enhancing Neutrophil Recruitment to Sites of Inflammation. J. Immunol..

[30] Yin H.-S., Li Y.-J., Jiang Z.-A., Liu S.-Y., Guo B.-Y., Wang T. (2014). Nicotine-induced ICAM-1 and VCAM-1 expression in mouse cardiac vascular endothelial cell via p38 MAPK signaling pathway. Anal. Quant. Cytopathol. Histopathol..

[31] Jeong Y., Du R., Zhu X., Yin S., Wang J., Cui H., Cao W., Lowenstein C.J. (2014). Histone deacetylase isoforms regulate innate immune responses by deacetylating mitogen-activated protein kinase phosphatase. J. Leukoc. Biol..

[32] Wang D., Wang Z., Zhang L., Wang Y. (2017). Roles of Cells from the Arterial Vessel Wall in Atherosclerosis. Mediat. Inflamm..

[33] Pollheimer J., Bodin J., Sundnes O., Edelmann R.J., Skånland S.S., Sponheim J., Brox M.J., Sundlisaeter E., Loos T., Vatn M. (2013). Interleukin-33 drives a proinflamma-tory endothelial activation that selectively targets nonquiescent cells. Arterioscler. Thromb. Vasc. Biol..

[34] Arshad M.I., Guihard P., Danger Y., Noel G., Le Seyec J., Boutet M.-A., Richards C.D., L’Helgoualc’H A., Genet V., Lucas-Clerc C. (2015). Oncostatin M induces IL-33 expression in liver endothelial cells in mice and expands ST2+CD4+lymphocytes. Am. J. Physiol. Liver Physiol..

[35] Boisvert W.A. (2004). The participation of chemokines in atherosclerosis. Discov. Med..

[36] Henricks P.A., Nijkamp F.P. (1998). Pharmacological modulation of cell adhesion molecules. Eur. J. Pharmacol..

[37] Li M., Van Esch B.C., Wagenaar G.T., Garssen J., Folkerts G., Henricks P.A. (2018). Pro- and anti-inflammatory effects of short chain fatty acids on immune and endothelial cells. Eur. J. Pharmacol..

[38] Liu S.F., Malik A.B. (2006). NF-κB activation as a pathological mechanism of septic shock and inflammation. Am. J. Physiol. Lung Cell. Mol. Physiol..

[39] Hsuan C.-F., Hsu H.-F., Tseng W.-K., Lee T.-L., Wei Y.-F., Hsu K.-L., Wu C.-C., Houng J.-Y. (2015). Glossogyne tenuifolia ex-tract inhibits TNF-α-induced expression of adhesion molecules in human umbilical vein en dothelial cells via blocking the NF-kB signaling pathway. Molecules.

[40] Kunsch C., Rosen C.A. (1993). NF-kappa B subunit-specific regulation of the interleukin-8 promoter. Mol. Cell. Biol..

[41] Liang C.-J., Wang S.-H., Chen Y.-H., Chang S.-S., Hwang T.-L., Leu Y.-L., Tseng Y.-C., Li C.-Y., Chen Y.-L. (2011). Viscolin reduces VCAM-1 expression in TNF-α-treated endothelial cells via the JNK/NF-κB and ROS pathway. Free. Radic. Biol. Med..

[42] Wagner E.F., Nebreda Á.R. (2009). Signal integration by JNK and p38 MAPK pathways in cancer development. Nat. Rev. Cancer.

[43] Jaffe E.A., Nachman R.L., Becker C.G., Minick C.R. (1973). Culture of Human Endothelial Cells Derived from Umbilical Veins. Identification by Morphologic and Immunologic Criteria. J. Clin. Investig..

[44] Westenbroek R.E., Bischoff S., Fu Y., Maier S.K., Catterall W.A., Scheuer T. (2013). Localization of sodium channel subtypes in mouse ventricular myocytes using quantitative immunocytochemistry. J. Mol. Cell. Cardiol..

[45] Curtis M.J., Bond R.A., Spina D., Ahluwalia A., Alexander S.P.A., Giembycz M.A., Gilchrist A., Hoyer D., Insel P.A., Izzo A.A. (2015). Experimental design and analysis and their reporting: New guidance for publication in BJP. Br. J. Pharmacol..

